# Chloridobis[diphenyl­glyoximato(1–)-κ^2^ *N*,*N*′](1*H*-imidazole-κ*N* ^3^)cobalt(III) hemihydrate

**DOI:** 10.1107/S160053680804347X

**Published:** 2009-01-08

**Authors:** P. Meera, C. Revathi, A. Dayalan

**Affiliations:** a Department of Chemistry, Loyola College (Autonomous), Chennai 600 034, Tamil Nadu, India

## Abstract

The Co centre in the title compound, [Co(C_14_H_11_N_2_O_2_)_2_Cl(C_3_H_4_N_2_)]·0.5H_2_O, shows a slightly distorted octa­hedral coordination geometry. The glyoximate units of the mol­ecule are linked by O—H⋯O hydrogen bonds with the H atom almost in the middle of the two O atoms. The crystal packing is stabilized through inter­molecular N—H⋯O, N—H⋯N and O—H⋯Cl hydrogen bonds. The uncoordinated water mol­ecule shows half-occupation.

## Related literature

For related literature, see: Calleri *et al.* (1967 ▶); Gupta *et al.* (2001 ▶, 2004 ▶); Lopez *et al.* (1991 ▶); Mandal & Gupta (2005 ▶); Silverstein & Bassler (1984 ▶); Toscano *et al.* (1983 ▶).
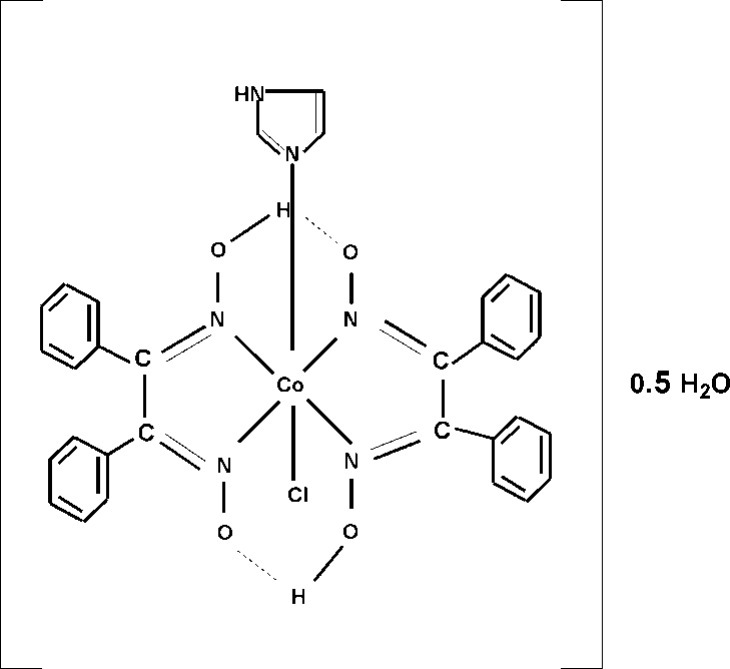


## Experimental

### 

#### Crystal data

[Co(C_14_H_11_N_2_O_2_)_2_Cl(C_3_H_4_N_2_)]·0.5H_2_O
*M* *_r_* = 649.97
Orthorhombic, 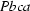

*a* = 19.1004 (11) Å
*b* = 12.0462 (7) Å
*c* = 26.9627 (18) Å
*V* = 6203.8 (7) Å^3^
*Z* = 8
Mo *K*α radiation
μ = 0.69 mm^−1^
*T* = 293 (2) K
0.30 × 0.20 × 0.20 mm

#### Data collection

Bruker Kappa APEXII CCD diffractometer
Absorption correction: multi-scan (*SADABS*; Bruker, 1999 ▶) *T*_min_ = 0.732, *T*_max_ = 0.850
28473 measured reflections
5282 independent reflections
3705 reflections with *I* > 2σ(*I*)
*R*_int_ = 0.051

#### Refinement

*R*[*F* ^2^ > 2σ(*F* ^2^)] = 0.040
*wR*(*F* ^2^) = 0.104
*S* = 1.06
5282 reflections
415 parameters
3 restraints
H atoms treated by a mixture of independent and constrained refinement
Δρ_max_ = 0.35 e Å^−3^
Δρ_min_ = −0.26 e Å^−3^

### 

Data collection: *APEX2* (Bruker, 2004 ▶); cell refinement: *APEX2* and *SAINT* (Bruker, 2004 ▶); data reduction: *SAINT* and *XPREP* (Bruker, 2004 ▶); program(s) used to solve structure: *SIR92* (Altomare *et al.*, 1993 ▶); program(s) used to refine structure: *SHELXL97* (Sheldrick, 2008 ▶); molecular graphics: *ORTEP-3* (Farrugia, 1997 ▶) and *Mercury* (Macrae *et al.*, 2006 ▶); software used to prepare material for publication: *SHELXL97*.

## Supplementary Material

Crystal structure: contains datablocks global, I. DOI: 10.1107/S160053680804347X/bt2831sup1.cif

Structure factors: contains datablocks I. DOI: 10.1107/S160053680804347X/bt2831Isup2.hkl

Additional supplementary materials:  crystallographic information; 3D view; checkCIF report

## Figures and Tables

**Table 1:** Hydrogen-bond geometry (Å, °)

*D*—H⋯*A*	*D*—H	H⋯*A*	*D*⋯*A*	*D*—H⋯*A*
O4—H2⋯O2	1.05 (4)	1.46 (4)	2.482 (3)	165 (3)
O3—H3⋯O1	1.07 (5)	1.39 (5)	2.456 (3)	174 (4)
N6—H6*A*⋯O2^i^	0.98 (4)	1.78 (4)	2.747 (4)	166 (3)
N6—H6*A*⋯N2^i^	0.98 (4)	2.50 (4)	3.326 (4)	141 (3)
O5—H5*B*⋯Cl1	0.946 (10)	2.69 (8)	3.331 (7)	126 (7)

Symmetry code: (i) 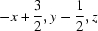.

**Fig. 1.**
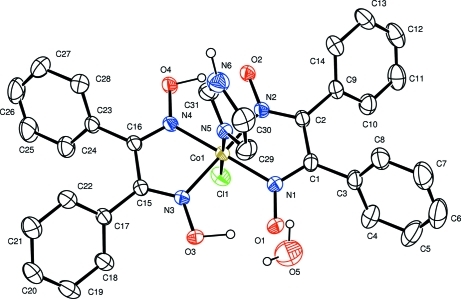
The ORTEP representation

**Fig. 2.**
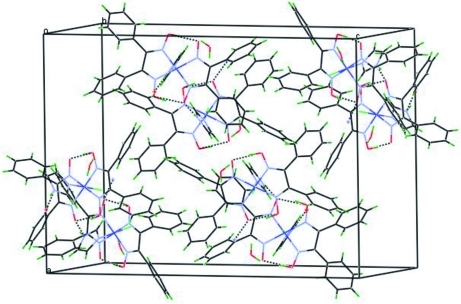
Packing of molecules in the unit cell. Hydrogen bonds are shown with dotted lines.

## supplementary crystallographic information

### Comment

The coordination geometry around cobalt is octahedral with the four nitrogen atoms of the diphenyl glyoximato ligand forming an approximate square plane. The bite angles N1—Co—N2 and N3—Co—N4 of the ligand are 81.40 (10)° and 80.32 (10)°, respectively. The coordinating chlorine and imidazole nitrogen [N5—Co1—Cl1 = 179.11 (7)°] are perpendicular to the equatorial plane composed by the four N atoms. The two glyoximate moieties are linked by strong O—H···O hydrogen bonds. Similar hydrogen bonds are found in nickel(II)glyoximate (Calleri, *et al.*, 1967).   The molecule is linked to its b-glide equivalent through a N—H···O hydrogen bond The water molecule forms a short O—H···Cl contact.

### Experimental

Cobaltous chloride hexahydrate was thoroughly ground and exposed to microwave for 30 s. The dehydrated salt was mixed with diphenylglyoxime in 1:2 molar ratio in acetone medium and was stirred for an hour (Toscano, *et al.*, 1983; Gupta, *et al.*, 2001). Since the dichloro complex of diphenyl glyoxime was non-isolable, the complex solution was as such refluxed with equimolar ratio of imidazole for six hours to get the title compound. The resulting brown mass was filtered, washed with ether and dried in a vaccuum desiccator. The complex was dissolved in ethanol and kept in a dark room for crystallization. Brown crystals of the complex appeared in three days. The elemental analysis data, obtained by analytical methods agree well with the theoretical data expected for the formula of the complex proposed: Anal%, (cald%): C, 62.07(62.57); H, 4.82(4.71); N, 14.50(14.13). The C=N stretching vibration of oxime in the complex was observed at 1629 cm^-1^ and the intra molecular hydrogen bonded OH around 3400 cm^-1^. A moderate peak around 1252 cm^-1^ may be assigned to the C=N—O stretching of the oxime. The peak around 537 cm^-1^ could be attributed to cobalt(III)-nitrogen stretching. The ^1^H NMR spectra of the complex in acetone-d_6_ shows three different signals corresponding to diphenyl glyoximate ring protons (Gupta, *et al.*, 2004; Lopez, *et al.*, 1991). The *ortho* H atoms of the ring shows a doublet at d= 7.4 p.p.m., the *meta* protons and the *para* proton give triplets at d = 7.6 and 7.9 p.p.m. respectively. The oxime –OH resonates at d=8.3 p.p.m.. The axial protons also appeared as multiplets along with phenyl protons at 7.2 and 7.4 p.p.m. as three proton signal (Silverstein & Bassler, 1984; Mandal & Gupta, 2005).

### Refinement

All the hydrogen atoms could be located in difference Fourier maps. Nevertheless, the phenyl H atoms were geometrically positioned  [C—H = 0.93 Å and U(H) = 1.2 U_eq_(O)] and were refined using a riding model. H atoms  bonded to O were refined isotropically with U(H) set to 1.2 U_eq_(O). For the water molecule the O-H distances were restrained to 0.95 (1)Å and the H···H distance to 1.55 (1)Å. Refinement of the water oxygen with full occupancy showed abnormally high displacement parameters. Hence, the site occupancy factor set to 0.5.

### Crystal data

**Table:**

[Co(C_14_H_11_N_2_O_2_)_2_Cl(C_3_H_4_N_2_)]·0.5H_2_O	*D*_x_ = 1.392 Mg m^−^^3^
*M**_r_* = 649.97	Mo *K*α radiation, λ = 0.71073 Å
Orthorhombic, *P**b**c**a*	Cell parameters from 5466 reflections
*a* = 19.1004 (11) Å	θ = 2.1–24.9°
*b* = 12.0462 (7) Å	µ = 0.69 mm^−^^1^
*c* = 26.9627 (18) Å	*T* = 293 K
*V* = 6203.8 (7) Å^3^	Needle, brown
*Z* = 8	0.30 × 0.20 × 0.20 mm
*F*(000) = 2680	

### Data collection

**Table:**

Bruker Kappa APEXII CCD diffractometer	5282 independent reflections
Radiation source: fine-focus sealed tube	3705 reflections with *I* > 2σ(*I*)
graphite	*R*_int_ = 0.051
ω and φ scans	θ_max_ = 24.7°, θ_min_ = 2.1°
Absorption correction: multi-scan (*SADABS*; Bruker, 1999)	*h* = −20→22
*T*_min_ = 0.732, *T*_max_ = 0.850	*k* = −14→13
28473 measured reflections	*l* = −31→18

### Refinement

**Table:**

Refinement on *F*^2^	Primary atom site location: structure-invariant direct methods
Least-squares matrix: full	Secondary atom site location: difference Fourier map
*R*[*F*^2^ > 2σ(*F*^2^)] = 0.040	Hydrogen site location: inferred from neighbouring sites
*wR*(*F*^2^) = 0.104	H atoms treated by a mixture of independent and constrained refinement
*S* = 1.06	*w* = 1/[σ^2^(*F*_o_^2^) + (0.0434*P*)^2^ + 2.9454*P*] where *P* = (*F*_o_^2^ + 2*F*_c_^2^)/3
5282 reflections	(Δ/σ)_max_ = 0.001
415 parameters	Δρ_max_ = 0.35 e Å^−^^3^
3 restraints	Δρ_min_ = −0.26 e Å^−^^3^

### Special details

**Table:**

Geometry. All e.s.d.'s (except the e.s.d. in the dihedral angle between two l.s. planes) are estimated using the full covariance matrix. The cell e.s.d.'s are taken into account individually in the estimation of e.s.d.'s in distances, angles and torsion angles; correlations between e.s.d.'s in cell parameters are only used when they are defined by crystal symmetry. An approximate (isotropic) treatment of cell e.s.d.'s is used for estimating e.s.d.'s involving l.s. planes.
Refinement. Refinement of *F*^2^ against ALL reflections. The weighted *R*-factor *wR* and goodness of fit *S* are based on *F*^2^, conventional *R*-factors *R* are based on *F*, with *F* set to zero for negative *F*^2^. The threshold expression of *F*^2^ > σ(*F*^2^) is used only for calculating *R*-factors(gt) *etc*. and is not relevant to the choice of reflections for refinement. *R*-factors based on *F*^2^ are statistically about twice as large as those based on *F*, and *R*- factors based on ALL data will be even larger.

### Fractional atomic coordinates and isotropic or equivalent isotropic displacement parameters (Å^2^)

**Table:**

	*x*	*y*	*z*	*U*_iso_*/*U*_eq_	Occ. (<1)
C1	0.63561 (15)	0.1880 (3)	0.00280 (11)	0.0409 (7)	
C2	0.70947 (14)	0.1624 (2)	0.01198 (11)	0.0381 (7)	
C3	0.60509 (15)	0.2080 (3)	−0.04659 (11)	0.0464 (8)	
C4	0.56950 (19)	0.3038 (3)	−0.05731 (13)	0.0639 (10)	
H4	0.5658	0.3596	−0.0336	0.077*	
C5	0.5390 (2)	0.3174 (4)	−0.10368 (17)	0.0861 (14)	
H5	0.5154	0.3827	−0.1114	0.103*	
C6	0.5438 (2)	0.2339 (5)	−0.13802 (16)	0.0882 (15)	
H6	0.5224	0.2427	−0.1688	0.106*	
C7	0.5790 (2)	0.1389 (5)	−0.12817 (15)	0.0837 (13)	
H7	0.5823	0.0832	−0.1520	0.100*	
C8	0.61007 (18)	0.1258 (3)	−0.08218 (13)	0.0624 (10)	
H8	0.6346	0.0610	−0.0751	0.075*	
C9	0.76536 (15)	0.1743 (3)	−0.02548 (10)	0.0406 (7)	
C10	0.76866 (18)	0.2672 (3)	−0.05512 (12)	0.0575 (9)	
H10	0.7342	0.3215	−0.0526	0.069*	
C11	0.8230 (2)	0.2803 (4)	−0.08864 (14)	0.0707 (11)	
H11	0.8251	0.3434	−0.1084	0.085*	
C12	0.8733 (2)	0.2007 (4)	−0.09260 (14)	0.0701 (11)	
H12	0.9094	0.2094	−0.1154	0.084*	
C13	0.87115 (19)	0.1089 (4)	−0.06346 (14)	0.0664 (11)	
H13	0.9060	0.0554	−0.0660	0.080*	
C14	0.81727 (17)	0.0953 (3)	−0.03009 (12)	0.0529 (9)	
H14	0.8158	0.0320	−0.0104	0.064*	
C15	0.57331 (14)	0.0802 (2)	0.18684 (11)	0.0364 (7)	
C16	0.64821 (14)	0.0580 (2)	0.19665 (10)	0.0365 (7)	
C17	0.51700 (14)	0.0551 (3)	0.22257 (11)	0.0402 (7)	
C18	0.46087 (16)	0.1271 (3)	0.22873 (13)	0.0550 (9)	
H18	0.4589	0.1930	0.2108	0.066*	
C19	0.40809 (19)	0.1009 (4)	0.26147 (15)	0.0737 (12)	
H19	0.3707	0.1495	0.2657	0.088*	
C20	0.41012 (19)	0.0049 (4)	0.28760 (15)	0.0781 (13)	
H20	0.3743	−0.0119	0.3097	0.094*	
C21	0.46428 (19)	−0.0669 (4)	0.28165 (15)	0.0758 (12)	
H21	0.4651	−0.1331	0.2993	0.091*	
C22	0.51801 (17)	−0.0419 (3)	0.24951 (12)	0.0554 (9)	
H22	0.5553	−0.0909	0.2460	0.067*	
C23	0.67758 (14)	0.0249 (3)	0.24497 (11)	0.0434 (8)	
C24	0.66383 (18)	0.0875 (3)	0.28642 (13)	0.0623 (10)	
H24	0.6353	0.1499	0.2840	0.075*	
C25	0.6919 (2)	0.0584 (5)	0.33133 (14)	0.0855 (14)	
H25	0.6824	0.1008	0.3594	0.103*	
C26	0.7334 (2)	−0.0319 (5)	0.33480 (17)	0.0930 (16)	
H26	0.7528	−0.0507	0.3653	0.112*	
C27	0.7473 (2)	−0.0954 (4)	0.29465 (17)	0.0851 (14)	
H27	0.7752	−0.1582	0.2978	0.102*	
C28	0.71984 (18)	−0.0669 (3)	0.24888 (14)	0.0660 (10)	
H28	0.7299	−0.1096	0.2210	0.079*	
C29	0.57488 (16)	−0.0595 (3)	0.04757 (12)	0.0548 (9)	
H29	0.5386	−0.0170	0.0346	0.066*	
C30	0.58509 (18)	−0.1672 (3)	0.03882 (13)	0.0593 (9)	
H30	0.5582	−0.2130	0.0187	0.071*	
C31	0.66589 (16)	−0.1065 (3)	0.08821 (12)	0.0488 (8)	
H31	0.7053	−0.1042	0.1084	0.059*	
N1	0.59804 (12)	0.1815 (2)	0.04272 (9)	0.0426 (6)	
N2	0.72105 (11)	0.13202 (19)	0.05738 (9)	0.0361 (6)	
N3	0.56368 (11)	0.1196 (2)	0.14284 (9)	0.0383 (6)	
N4	0.68677 (11)	0.0763 (2)	0.15816 (8)	0.0382 (6)	
N5	0.62594 (11)	−0.0215 (2)	0.07856 (9)	0.0380 (6)	
N6	0.64288 (15)	−0.1966 (3)	0.06536 (11)	0.0552 (7)	
O1	0.52933 (10)	0.1998 (2)	0.04167 (8)	0.0542 (6)	
O2	0.78462 (9)	0.10740 (17)	0.07404 (7)	0.0425 (5)	
O3	0.49944 (10)	0.14234 (19)	0.12648 (8)	0.0520 (6)	
O4	0.75702 (10)	0.0645 (2)	0.16194 (8)	0.0496 (6)	
O5	0.5658 (4)	0.4699 (6)	0.0555 (3)	0.127 (2)	0.50
H5B	0.611 (2)	0.438 (7)	0.055 (4)	0.152*	0.50
H5A	0.535 (4)	0.425 (7)	0.074 (4)	0.152*	0.50
Co1	0.642761 (18)	0.12847 (3)	0.100084 (14)	0.03524 (13)	
Cl1	0.66393 (4)	0.30158 (7)	0.12458 (3)	0.0560 (2)	
H2	0.7759 (17)	0.087 (3)	0.1270 (14)	0.070 (11)*	
H3	0.509 (2)	0.169 (4)	0.0893 (17)	0.106 (15)*	
H6A	0.6632 (18)	−0.272 (3)	0.0650 (13)	0.075 (12)*	

### Atomic displacement parameters (Å^2^)

**Table:**

	*U*^11^	*U*^22^	*U*^33^	*U*^12^	*U*^13^	*U*^23^
C1	0.0429 (17)	0.0496 (19)	0.0303 (18)	−0.0003 (14)	0.0007 (14)	0.0066 (14)
C2	0.0378 (16)	0.0466 (18)	0.0301 (18)	−0.0019 (13)	0.0026 (13)	0.0013 (13)
C3	0.0405 (17)	0.065 (2)	0.0334 (19)	−0.0075 (16)	0.0008 (14)	0.0093 (16)
C4	0.074 (2)	0.067 (3)	0.050 (2)	−0.002 (2)	−0.0123 (19)	0.0156 (19)
C5	0.082 (3)	0.102 (4)	0.075 (3)	−0.002 (3)	−0.020 (2)	0.043 (3)
C6	0.080 (3)	0.138 (5)	0.046 (3)	−0.030 (3)	−0.014 (2)	0.015 (3)
C7	0.075 (3)	0.131 (4)	0.045 (3)	−0.014 (3)	−0.003 (2)	−0.012 (3)
C8	0.055 (2)	0.089 (3)	0.043 (2)	0.0001 (19)	0.0009 (17)	−0.003 (2)
C9	0.0409 (16)	0.0508 (19)	0.0301 (17)	−0.0089 (15)	0.0017 (13)	−0.0025 (15)
C10	0.058 (2)	0.066 (2)	0.048 (2)	−0.0061 (18)	0.0019 (17)	0.0103 (18)
C11	0.080 (3)	0.080 (3)	0.052 (3)	−0.028 (2)	0.011 (2)	0.013 (2)
C12	0.059 (2)	0.100 (3)	0.052 (3)	−0.027 (2)	0.0202 (19)	−0.009 (2)
C13	0.058 (2)	0.084 (3)	0.057 (3)	−0.001 (2)	0.0203 (19)	−0.011 (2)
C14	0.059 (2)	0.058 (2)	0.042 (2)	−0.0033 (17)	0.0129 (16)	−0.0043 (16)
C15	0.0348 (15)	0.0451 (18)	0.0293 (17)	0.0025 (13)	0.0024 (12)	0.0010 (13)
C16	0.0341 (15)	0.0463 (18)	0.0293 (17)	0.0019 (13)	0.0021 (13)	−0.0015 (13)
C17	0.0342 (16)	0.054 (2)	0.0329 (17)	0.0007 (14)	0.0046 (13)	0.0020 (15)
C18	0.0485 (19)	0.060 (2)	0.057 (2)	0.0096 (17)	0.0165 (16)	0.0085 (17)
C19	0.054 (2)	0.090 (3)	0.078 (3)	0.019 (2)	0.031 (2)	0.012 (2)
C20	0.057 (2)	0.100 (3)	0.077 (3)	0.007 (2)	0.034 (2)	0.029 (3)
C21	0.061 (2)	0.085 (3)	0.082 (3)	0.006 (2)	0.028 (2)	0.033 (2)
C22	0.0460 (19)	0.063 (2)	0.057 (2)	0.0088 (16)	0.0122 (16)	0.0135 (19)
C23	0.0312 (15)	0.070 (2)	0.0294 (18)	0.0010 (15)	−0.0004 (13)	0.0039 (15)
C24	0.062 (2)	0.088 (3)	0.038 (2)	0.0011 (19)	−0.0025 (17)	−0.0001 (19)
C25	0.079 (3)	0.142 (4)	0.035 (2)	0.002 (3)	−0.011 (2)	−0.002 (2)
C26	0.069 (3)	0.161 (5)	0.049 (3)	−0.001 (3)	−0.014 (2)	0.037 (3)
C27	0.068 (3)	0.120 (4)	0.068 (3)	0.028 (2)	−0.005 (2)	0.036 (3)
C28	0.057 (2)	0.088 (3)	0.053 (2)	0.016 (2)	0.0057 (18)	0.015 (2)
C29	0.0425 (19)	0.067 (3)	0.054 (2)	−0.0026 (17)	−0.0135 (16)	0.0037 (18)
C30	0.057 (2)	0.063 (3)	0.057 (2)	−0.0122 (18)	−0.0107 (18)	−0.0093 (19)
C31	0.0416 (17)	0.058 (2)	0.047 (2)	0.0045 (16)	−0.0048 (15)	−0.0061 (16)
N1	0.0347 (13)	0.0580 (17)	0.0350 (15)	0.0065 (12)	−0.0001 (11)	0.0056 (12)
N2	0.0299 (12)	0.0463 (14)	0.0320 (14)	−0.0004 (11)	0.0003 (10)	−0.0002 (11)
N3	0.0294 (12)	0.0527 (15)	0.0330 (15)	0.0063 (11)	0.0025 (10)	0.0035 (12)
N4	0.0280 (12)	0.0570 (16)	0.0295 (14)	0.0033 (11)	−0.0008 (10)	−0.0025 (11)
N5	0.0324 (12)	0.0502 (15)	0.0316 (14)	0.0044 (11)	−0.0007 (11)	0.0012 (12)
N6	0.0563 (18)	0.0526 (19)	0.057 (2)	0.0056 (15)	0.0025 (14)	−0.0056 (15)
O1	0.0346 (11)	0.0849 (17)	0.0429 (14)	0.0141 (11)	−0.0007 (10)	0.0164 (12)
O2	0.0293 (10)	0.0635 (14)	0.0347 (12)	0.0020 (9)	0.0022 (9)	0.0051 (10)
O3	0.0279 (10)	0.0848 (17)	0.0432 (14)	0.0143 (10)	0.0013 (9)	0.0154 (12)
O4	0.0258 (10)	0.0886 (17)	0.0343 (13)	0.0043 (10)	0.0010 (9)	0.0078 (12)
O5	0.144 (7)	0.109 (6)	0.127 (7)	0.037 (5)	0.001 (5)	0.011 (4)
Co1	0.0288 (2)	0.0490 (2)	0.0280 (2)	0.00340 (18)	0.00147 (16)	0.00213 (18)
Cl1	0.0571 (5)	0.0530 (5)	0.0580 (6)	−0.0016 (4)	0.0119 (4)	−0.0087 (4)

### Geometric parameters (Å, °)

**Table:**

C1—N1	1.296 (4)	C21—C22	1.376 (4)
C1—C2	1.465 (4)	C21—H21	0.9300
C1—C3	1.473 (4)	C22—H22	0.9300
C2—N2	1.296 (3)	C23—C28	1.373 (5)
C2—C9	1.477 (4)	C23—C24	1.374 (5)
C3—C4	1.370 (5)	C24—C25	1.370 (5)
C3—C8	1.382 (5)	C24—H24	0.9300
C4—C5	1.389 (5)	C25—C26	1.348 (6)
C4—H4	0.9300	C25—H25	0.9300
C5—C6	1.370 (6)	C26—C27	1.352 (6)
C5—H5	0.9300	C26—H26	0.9300
C6—C7	1.354 (6)	C27—C28	1.384 (5)
C6—H6	0.9300	C27—H27	0.9300
C7—C8	1.384 (5)	C28—H28	0.9300
C7—H7	0.9300	C29—C30	1.333 (5)
C8—H8	0.9300	C29—N5	1.364 (4)
C9—C10	1.377 (4)	C29—H29	0.9300
C9—C14	1.380 (4)	C30—N6	1.362 (4)
C10—C11	1.385 (5)	C30—H30	0.9300
C10—H10	0.9300	C31—N5	1.304 (4)
C11—C12	1.362 (6)	C31—N6	1.324 (4)
C11—H11	0.9300	C31—H31	0.9300
C12—C13	1.357 (5)	N1—O1	1.331 (3)
C12—H12	0.9300	N1—Co1	1.879 (2)
C13—C14	1.377 (5)	N2—O2	1.328 (3)
C13—H13	0.9300	N2—Co1	1.888 (2)
C14—H14	0.9300	N3—O3	1.332 (3)
C15—N3	1.291 (3)	N3—Co1	1.903 (2)
C15—C17	1.475 (4)	N4—O4	1.353 (3)
C15—C16	1.479 (4)	N4—Co1	1.885 (2)
C16—N4	1.291 (3)	N5—Co1	1.924 (2)
C16—C23	1.474 (4)	N6—H6A	0.98 (4)
C17—C22	1.376 (4)	O1—H3	1.39 (5)
C17—C18	1.389 (4)	O2—H2	1.46 (4)
C18—C19	1.377 (4)	O3—H3	1.07 (5)
C18—H18	0.9300	O4—H2	1.05 (4)
C19—C20	1.355 (5)	O5—H5B	0.946 (10)
C19—H19	0.9300	O5—H5A	0.946 (10)
C20—C21	1.358 (5)	Co1—Cl1	2.2244 (9)
C20—H20	0.9300		
			
N1—C1—C2	112.3 (3)	C28—C23—C24	119.5 (3)
N1—C1—C3	122.8 (3)	C28—C23—C16	120.7 (3)
C2—C1—C3	124.6 (3)	C24—C23—C16	119.8 (3)
N2—C2—C1	112.5 (2)	C25—C24—C23	120.2 (4)
N2—C2—C9	123.4 (3)	C25—C24—H24	119.9
C1—C2—C9	124.0 (3)	C23—C24—H24	119.9
C4—C3—C8	119.4 (3)	C26—C25—C24	119.9 (4)
C4—C3—C1	121.6 (3)	C26—C25—H25	120.1
C8—C3—C1	118.9 (3)	C24—C25—H25	120.1
C3—C4—C5	119.8 (4)	C25—C26—C27	121.0 (4)
C3—C4—H4	120.1	C25—C26—H26	119.5
C5—C4—H4	120.1	C27—C26—H26	119.5
C6—C5—C4	119.6 (4)	C26—C27—C28	120.0 (4)
C6—C5—H5	120.2	C26—C27—H27	120.0
C4—C5—H5	120.2	C28—C27—H27	120.0
C7—C6—C5	121.4 (4)	C23—C28—C27	119.4 (4)
C7—C6—H6	119.3	C23—C28—H28	120.3
C5—C6—H6	119.3	C27—C28—H28	120.3
C6—C7—C8	119.0 (4)	C30—C29—N5	109.3 (3)
C6—C7—H7	120.5	C30—C29—H29	125.3
C8—C7—H7	120.5	N5—C29—H29	125.3
C3—C8—C7	120.7 (4)	C29—C30—N6	106.2 (3)
C3—C8—H8	119.6	C29—C30—H30	126.9
C7—C8—H8	119.6	N6—C30—H30	126.9
C10—C9—C14	118.4 (3)	N5—C31—N6	110.9 (3)
C10—C9—C2	120.6 (3)	N5—C31—H31	124.5
C14—C9—C2	120.9 (3)	N6—C31—H31	124.5
C9—C10—C11	120.4 (4)	C1—N1—O1	121.2 (2)
C9—C10—H10	119.8	C1—N1—Co1	116.91 (19)
C11—C10—H10	119.8	O1—N1—Co1	121.47 (18)
C12—C11—C10	120.0 (4)	C2—N2—O2	122.6 (2)
C12—C11—H11	120.0	C2—N2—Co1	116.56 (19)
C10—C11—H11	120.0	O2—N2—Co1	120.84 (17)
C13—C12—C11	120.5 (3)	C15—N3—O3	120.7 (2)
C13—C12—H12	119.8	C15—N3—Co1	117.66 (18)
C11—C12—H12	119.8	O3—N3—Co1	121.26 (18)
C12—C13—C14	119.9 (4)	C16—N4—O4	119.1 (2)
C12—C13—H13	120.1	C16—N4—Co1	118.04 (19)
C14—C13—H13	120.1	O4—N4—Co1	122.67 (17)
C13—C14—C9	120.9 (3)	C31—N5—C29	106.1 (3)
C13—C14—H14	119.6	C31—N5—Co1	125.4 (2)
C9—C14—H14	119.6	C29—N5—Co1	128.3 (2)
N3—C15—C17	124.9 (2)	C31—N6—C30	107.5 (3)
N3—C15—C16	111.6 (2)	C31—N6—H6A	129 (2)
C17—C15—C16	123.5 (3)	C30—N6—H6A	124 (2)
N4—C16—C23	122.7 (2)	N1—O1—H3	101.9 (17)
N4—C16—C15	112.2 (2)	N2—O2—H2	105.3 (13)
C23—C16—C15	125.1 (2)	N3—O3—H3	102 (2)
C22—C17—C18	118.6 (3)	N4—O4—H2	104.2 (18)
C22—C17—C15	120.6 (3)	H5B—O5—H5A	110.0 (17)
C18—C17—C15	120.9 (3)	N1—Co1—N4	179.23 (11)
C19—C18—C17	119.9 (3)	N1—Co1—N2	81.39 (10)
C19—C18—H18	120.0	N4—Co1—N2	99.27 (10)
C17—C18—H18	120.0	N1—Co1—N3	99.02 (10)
C20—C19—C18	120.6 (3)	N4—Co1—N3	80.33 (10)
C20—C19—H19	119.7	N2—Co1—N3	178.07 (10)
C18—C19—H19	119.7	N1—Co1—N5	89.71 (11)
C19—C20—C21	120.3 (3)	N4—Co1—N5	90.69 (10)
C19—C20—H20	119.9	N2—Co1—N5	88.25 (10)
C21—C20—H20	119.9	N3—Co1—N5	89.86 (10)
C20—C21—C22	120.2 (4)	N1—Co1—Cl1	90.47 (8)
C20—C21—H21	119.9	N4—Co1—Cl1	89.13 (8)
C22—C21—H21	119.9	N2—Co1—Cl1	90.91 (7)
C17—C22—C21	120.5 (3)	N3—Co1—Cl1	90.97 (8)
C17—C22—H22	119.8	N5—Co1—Cl1	179.11 (7)
C21—C22—H22	119.8		
			
N1—C1—C2—N2	4.0 (4)	C17—C15—N3—O3	0.9 (4)
C3—C1—C2—N2	−170.2 (3)	C16—C15—N3—O3	178.3 (2)
N1—C1—C2—C9	−173.5 (3)	C17—C15—N3—Co1	−172.4 (2)
C3—C1—C2—C9	12.3 (5)	C16—C15—N3—Co1	5.0 (3)
N1—C1—C3—C4	62.3 (4)	C23—C16—N4—O4	0.2 (4)
C2—C1—C3—C4	−124.1 (4)	C15—C16—N4—O4	177.1 (2)
N1—C1—C3—C8	−114.9 (4)	C23—C16—N4—Co1	−175.5 (2)
C2—C1—C3—C8	58.7 (4)	C15—C16—N4—Co1	1.4 (3)
C8—C3—C4—C5	−0.1 (5)	N6—C31—N5—C29	−0.4 (4)
C1—C3—C4—C5	−177.3 (3)	N6—C31—N5—Co1	−175.6 (2)
C3—C4—C5—C6	1.1 (6)	C30—C29—N5—C31	−0.3 (4)
C4—C5—C6—C7	−1.4 (7)	C30—C29—N5—Co1	174.7 (2)
C5—C6—C7—C8	0.7 (7)	N5—C31—N6—C30	0.9 (4)
C4—C3—C8—C7	−0.6 (5)	C29—C30—N6—C31	−1.0 (4)
C1—C3—C8—C7	176.6 (3)	C1—N1—Co1—N4	154 (9)
C6—C7—C8—C3	0.3 (6)	O1—N1—Co1—N4	−33 (9)
N2—C2—C9—C10	−132.9 (3)	C1—N1—Co1—N2	4.7 (2)
C1—C2—C9—C10	44.3 (4)	O1—N1—Co1—N2	177.4 (2)
N2—C2—C9—C14	44.1 (4)	C1—N1—Co1—N3	−173.4 (2)
C1—C2—C9—C14	−138.7 (3)	O1—N1—Co1—N3	−0.7 (2)
C14—C9—C10—C11	0.0 (5)	C1—N1—Co1—N5	−83.6 (2)
C2—C9—C10—C11	177.1 (3)	O1—N1—Co1—N5	89.1 (2)
C9—C10—C11—C12	0.3 (6)	C1—N1—Co1—Cl1	95.5 (2)
C10—C11—C12—C13	−0.8 (6)	O1—N1—Co1—Cl1	−91.7 (2)
C11—C12—C13—C14	0.9 (6)	C16—N4—Co1—N1	33 (9)
C12—C13—C14—C9	−0.5 (6)	O4—N4—Co1—N1	−142 (9)
C10—C9—C14—C13	0.1 (5)	C16—N4—Co1—N2	−177.2 (2)
C2—C9—C14—C13	−177.0 (3)	O4—N4—Co1—N2	7.3 (2)
N3—C15—C16—N4	−4.0 (4)	C16—N4—Co1—N3	0.9 (2)
C17—C15—C16—N4	173.4 (3)	O4—N4—Co1—N3	−174.6 (2)
N3—C15—C16—C23	172.8 (3)	C16—N4—Co1—N5	−88.8 (2)
C17—C15—C16—C23	−9.8 (5)	O4—N4—Co1—N5	95.6 (2)
N3—C15—C17—C22	136.0 (3)	C16—N4—Co1—Cl1	92.1 (2)
C16—C15—C17—C22	−41.1 (4)	O4—N4—Co1—Cl1	−83.5 (2)
N3—C15—C17—C18	−42.1 (5)	C2—N2—Co1—N1	−2.2 (2)
C16—C15—C17—C18	140.8 (3)	O2—N2—Co1—N1	176.0 (2)
C22—C17—C18—C19	0.4 (5)	C2—N2—Co1—N4	178.2 (2)
C15—C17—C18—C19	178.5 (3)	O2—N2—Co1—N4	−3.6 (2)
C17—C18—C19—C20	−0.4 (6)	C2—N2—Co1—N3	100 (3)
C18—C19—C20—C21	−0.2 (7)	O2—N2—Co1—N3	−82 (3)
C19—C20—C21—C22	0.9 (7)	C2—N2—Co1—N5	87.8 (2)
C18—C17—C22—C21	0.3 (5)	O2—N2—Co1—N5	−94.1 (2)
C15—C17—C22—C21	−177.9 (3)	C2—N2—Co1—Cl1	−92.5 (2)
C20—C21—C22—C17	−0.9 (6)	O2—N2—Co1—Cl1	85.63 (19)
N4—C16—C23—C28	−55.4 (4)	C15—N3—Co1—N1	176.9 (2)
C15—C16—C23—C28	128.1 (3)	O3—N3—Co1—N1	3.7 (2)
N4—C16—C23—C24	123.3 (3)	C15—N3—Co1—N4	−3.5 (2)
C15—C16—C23—C24	−53.2 (4)	O3—N3—Co1—N4	−176.7 (2)
C28—C23—C24—C25	−0.2 (5)	C15—N3—Co1—N2	75 (3)
C16—C23—C24—C25	−178.8 (3)	O3—N3—Co1—N2	−99 (3)
C23—C24—C25—C26	0.2 (6)	C15—N3—Co1—N5	87.2 (2)
C24—C25—C26—C27	−0.8 (7)	O3—N3—Co1—N5	−86.0 (2)
C25—C26—C27—C28	1.3 (7)	C15—N3—Co1—Cl1	−92.4 (2)
C24—C23—C28—C27	0.7 (5)	O3—N3—Co1—Cl1	94.3 (2)
C16—C23—C28—C27	179.3 (3)	C31—N5—Co1—N1	149.3 (3)
C26—C27—C28—C23	−1.2 (6)	C29—N5—Co1—N1	−24.8 (3)
N5—C29—C30—N6	0.8 (4)	C31—N5—Co1—N4	−31.4 (3)
C2—C1—N1—O1	−178.7 (3)	C29—N5—Co1—N4	154.5 (3)
C3—C1—N1—O1	−4.4 (5)	C31—N5—Co1—N2	67.9 (3)
C2—C1—N1—Co1	−5.9 (3)	C29—N5—Co1—N2	−106.2 (3)
C3—C1—N1—Co1	168.4 (2)	C31—N5—Co1—N3	−111.7 (3)
C1—C2—N2—O2	−178.4 (2)	C29—N5—Co1—N3	74.2 (3)
C9—C2—N2—O2	−0.9 (4)	C31—N5—Co1—Cl1	47 (5)
C1—C2—N2—Co1	−0.3 (3)	C29—N5—Co1—Cl1	−127 (5)
C9—C2—N2—Co1	177.2 (2)		

### Hydrogen-bond geometry (Å, °)

**Table:**

*D*—H···*A*	*D*—H	H···*A*	*D*···*A*	*D*—H···*A*
O4—H2···O2	1.05 (4)	1.46 (4)	2.482 (3)	165 (3)
O3—H3···O1	1.07 (5)	1.39 (5)	2.456 (3)	174 (4)
N6—H6A···O2^i^	0.98 (4)	1.78 (4)	2.747 (4)	166 (3)
N6—H6A···N2^i^	0.98 (4)	2.50 (4)	3.326 (4)	141 (3)
O5—H5B···Cl1	0.95 (1)	2.69 (8)	3.331 (7)	126 (7)

Symmetry codes: (i) −*x*+3/2, *y*−1/2, *z*.
